# The best interests of the child and the return of results in genetic research: international comparative perspectives

**DOI:** 10.1186/1472-6939-15-72

**Published:** 2014-10-04

**Authors:** Ma’n H Zawati, David Parry, Bartha Maria Knoppers

**Affiliations:** Centre of Genomics and Policy, McGill University, 740 Dr. Penfield Avenue, Suite 5200, Montreal, Quebec H3A 0G1 Canada

**Keywords:** Return of research results, Best interests of the child, Paediatrics, Genomic research, Convention on the Rights of the Child, Children’s rights

## Abstract

**Background:**

Paediatric genomic research raises particularly challenging questions on whether and under what circumstances to return research results. In the paediatric context, decision-making is guided by the best interests of the child framework, as enshrined in the 1989 international *Convention on the Rights of the Child*. According to this Convention*,* rights and responsibilities are shared between children, parents, researchers, and the state. These "relational" obligations are further complicated in the context of genetic research.

**Discussion:**

A comparative review of international, regional and national documents on the return of research results reveals that there is a dearth of normative documents in the paediatric context. The best interests of the child framework is increasingly complicated by a growing appreciation of pediatric autonomy and the development thereof; parental rights (particularly when parents are affected by the genomic information of their children); and the right not to know.

**Summary:**

This comparative analysis reveals that policy-makers and legislators have responded to the above challenges in different ways. Nevertheless, in Europe as well as in Canada, there is an emerging trend towards making the return of certain results mandatory in the paediatric context, should this course of action prove to be in the best interests of the child.

## Background

An "individual research result" is defined as "a finding concerning an individual research participant that has potential health or reproductive importance or personal utility and is discovered in the course of research […] in meeting the project’s research aims" [1]. In recent years, discussions surrounding the topic of the return of such results in genetic research have mainly focused on whether individual findings should be returned to participants and, if so, under what conditions [2]. The increasing availability and affordability of next-generation sequencing has made this debate all the more pressing [3].

That being said, little attention has been given to the challenging issue of return of results in *paediatric* genetic research, or to the complex dynamics of decision-making in this context. Indeed, while the ‘best interests of the child’ (‘BIC’) framework remains the foundation for decisions concerning children in research, these decisions are shared between 1) parents, who, as a matter of law, are authorized to speak for their children, guide their actions and determine their healthcare; 2) researchers, who have an obligation to conduct research according to professional norms; 3) children, whose views become increasingly important as they mature; and finally, 4) the State, which has the power to intervene in cases of neglect and to legislate on matters relating to the participation of children in research. In the case of paediatric genetics, where research results may not be actionable during childhood or may have implications for parents, siblings and other relatives, the best interests of the child framework is all the more complicated. What role do the aforementioned stakeholders play in the decision to return paediatric research findings? What are the general principles that guide such decision-making processes? What are the responsibilities of the various actors involved?

Despite the fact that children have been involved in medical research for hundreds of years, pediatric participation has been consistently overlooked in guidelines and policy-related documents [4]. In recent years, however, the international community has reached a consensus [4] on the importance of including children in research. Accordingly, this text will undertake a comparative analysis of international norms relating to the return of results in paediatric genomic research. First, this text will examine general principles as enshrined in the 1989 *Convention on the Rights of the Child* [5] ("CRC") (Section II.A). Second, it will review principles in regional (European) normative documents as well as professional norms (Section II.B). These norms, when adopted by professional bodies, often play a role in determining standard of care. Findings from Sections II.A and II.B will allow us to comprehend how these principles and professional norms are applied to the return of research results in paediatrics for select common and civil law countries (UK, France, the Netherlands, Spain and Canada)^a^ (Section II.C). Finally, we return to our international comparison in order to critically evaluate the future of the return of results in the context of paediatric research (Section II.D).

### Methodology

The international documents referenced in this text were collected using the HumGen International Database ( http://www.humgen.org), a database of international, national, and regional guidelines and policies specific to human genetic research. In order to narrow our search, the keywords "minor/child" and "communication of results" were used to identify those documents that were most relevant and important. Keywords such as "research result" or "incidental findings" were not available. All organizations were selected, and no limitations were set as to jurisdiction. This provided a large selection of documents. Only English documents dating from 1990 to 2013 were queried. This search generated 145 results, of which approximately 20 were selected based on their level of relevance to this text.

Case law was retrieved using Quicklaw, a legal database. For the purposes of our research, the keywords "research" and "best interests of the child" were used. This search generated 933 results; these results were then narrowed based on their relevance to the best interests of the child in medicine. Legal doctrine was retrieved using HeinOnline, a database of legal journals. The keywords "return of results," "genetic research" and "children" were used to facilitate the search process. This search yielded 25 results.

## Discussion

### International norms: general principles

There is a dearth of international normative literature on return of results in the paediatric context [6]. Existing policies and/or guidelines are primarily concerned with adult-level participation, and are extremely broad in both scope and application [4]. That being said, however, several important principles can be distilled from these normative documents that are highly relevant to the issue of return of results in paediatric research. For example, UNESCO’s 1997 *Universal Declaration on the Human Genome and Human Rights* sets out ‘[t]he right of each individual to decide whether or not to be informed of the results of genetic examination and the resulting consequences should be respected’ [7], despite the fact that it does not explore the nature of this right in the paediatric context.

In international law, the BIC is the central ethos for decisions concerning children participating in genomic research. Consequently, the CRC can be viewed as a "cornerstone" document; with the dual distinction of (1) having been unanimously approved by the UN’s General Assembly; and (2) being the most universally ratified UN treaty to date [8], the CRC offers a comprehensive statement on paediatric human rights. Article 3 of the CRC enshrines the BIC as a general principle, which has since been reinforced by a number of international normative documents.

The CRC also enshrines other important paediatric rights. Notably, for our purposes, these include the child’s ‘right to be heard’ [5] and right to enjoy ‘the highest attainable standard of health’ [5]. Though the CRC is not specific to paediatric research (and, in fact, this paper acknowledges that research is fundamentally different from clinical care), the driving spirit of this convention can be applied to the debate surrounding paediatric research. In point of fact, since the CRC came into force, scholarship on this convention has focused on the rights of autonomy and participation in the paediatric context, and decisions affecting these rights [9].

Importantly, the rights spelled out in the CRC exist ‘independently of […] parents and are enforceable by children over against their parents through the medium of the state’ [10]. This is significant both because it has altered the rights and responsibilities of those who share in decision-making surrounding children (*i.e*., parents, researchers, children, and the State), but also because children are seen to have independent legal rights (albeit usually exercised via a court-appointed tutor). Moreover, this duality begs the question of how to balance those rights and responsibilities under the BIC framework. For the purposes of this text, we will use the CRC’s definition of ‘child’, which refers to ‘every human being below the age of eighteen years unless under the law applicable to the child, majority is attained earlier’ [5].

#### The best interests of the child: definition and scope

Article 3 of the CRC states:

In all actions concerning children, whether undertaken by public or private social welfare institutions, courts of law, administrative authorities or legislative bodies, the best interests of the child shall be a primary consideration [5].

While this article emphasizes the paramountcy of the BIC, it is necessarily broad. Nonetheless, the CRC provides some indication of the principle’s overall scope. Indeed, this article establishes the BIC as a general principle guiding the interpretation of the entire CRC. In other words, when taking measures under article 24 (the right of the child to the enjoyment of the highest attainable standard of health) for example, States Parties to the CRC must consider whether a decision or policy satisfies the requirements outlined in article 3.

However, the interpretation of article 3 calls for a nuanced approach, with particular attention to wording. The use of the term ‘primary’ implies that the BIC principle is ‘not the only factor to be considered in the actions of institutions, authorities and administration’ [11]. Additionally, article 3 refers to the BIC as ‘a primary’ (and not ‘the primary’) consideration. Therefore, the BIC should be ‘among the first aspects to be considered and…given considerable weight in all decisions affecting children’ [12]. The World Medical Association’s *Declaration of Ottawa on Child Health* [13] adopts a similar approach: ‘[t]he best interests of the child shall be the primary consideration in the provision of health care’ [13] (emphasis added).

In 2008, the Council of Europe’s Commissioner for Human Rights tentatively defined the BIC principle as ‘the sum total of the norms in the CRC’ [12]. Thus, a decision is held to be in the best interests of the child if it most effectively applies the rights contained in the CRC. It requires the use of ‘good and reasonable options’ [14] that safeguard the rights of children to the maximum extent possible [15]. Furthermore, the BIC principle requires that children be placed at the centre of the decision-making process and that policies and strategies ‘be adapted to children’s rights and needs’ [16].

If the BIC principle is to inform paediatric genomic research, then it must favour the most effective application of the rights of the child. This means utilizing all ‘good and reasonable options’ to safeguard – and indeed assist in the exercise of – the rights of paediatric participants. However, the substantive content of these rights and how they are balanced against the best interests of the child remains an open question in genomic research. After all, though the CRC states that the BIC is a primary consideration, it is not the *only* consideration – children possess other rights that must coexist with the BIC principle. We now turn to some of these other rights.

#### The right to be heard: elements to consider

The UN Committee on the Rights of the Child (‘UN Committee’) views the right to be heard as a general principle, guiding the interpretation and implementation of all other paediatric rights [11]. Article 12, which codified this right for the first time, reads:

1. States Parties shall assure to the child who is capable of forming his or her own views the right to express those views freely in all matters affecting the child, the views of the child being given due weight in accordance with the age and maturity of the child [5].

Unlike article 3, this provision contains several specific criteria that must also be satisfied during the course of paediatric decision-making. Recall also that this right exists independent of parents, and consequently can be exercised independently of same [10].

As soon as children become capable of forming their own views, they should be given the opportunity to express them. As children mature, they should be granted a higher degree of participation. In this same vein, the Organization for Economic Cooperation and Development’s *Guidelines for Human Biobanks and Genetic Research Databases* recommends finding ‘ways in which minor[s] can play a more active role’ ‘in light of [their] age and autonomy’ [17]. This view appears to be supported by the UN Committee [11] and by previous versions of article 3, which included ‘the right of the child to be heard in relation to his or her best interests’ [18].

The right to be heard rests on the evaluation of a child’s mental capacity. In this regard, the UN Committee refers to an obligation of States Parties to ‘assess the capacity of the child to form an autonomous opinion to the greatest extent possible’ [11]. The Committee insists, however, that there be a presumption that children have the capacity to form their own views [11]. Thus, the right to be heard should not be linked to the child’s age, but to the acquisition of necessary intellectual, cognitive, social, and emotional skills. Children do not need to fully understand a complex issue (such as genomic research) to be able to express their opinions. As mentioned earlier, States Parties can determine the age of majority by law, and may also prescribe a specific age for participation in research.

Article 12 also requires that children be able to express their views ‘freely’. This freedom of expression comprises three criteria. First, children should be able to decide whether or not to exercise their rights [11]. Second, the right to be heard should be exercised without undue influence or pressure from family members and/or society. Third, children must have access to and receive complete information in accordance with articles 13 (freedom to seek information) and 17 (access to information) of the CRC [19]. According to the UN Committee:

Physicians and health-care facilities should provide clear and accessible information to children on their rights concerning their participation in paediatric research and clinical trials. They have to be informed about the research, so that their informed consent can be obtained in addition to other procedural safeguards [11].

Also, children’s views must be given due weight in accordance with their age and maturity.^b^ In the context of adolescent health, the UN Committee even recommends that sufficiently mature adolescents be given the right to consent to their own health care, and that parents should only be informed if such disclosure proves to be in the best interests of the child [20]. The UN Committee did not address the issue of whether a greater degree of ‘maturity’ is required for participation in research. Whereas clinical care provides therapeutic benefit to individual patients, research is intended to produce generalizable results for society as a whole. In view of the foregoing, it may be possible to infer a higher degree of maturity for paediatric participation in research – the better to avoid "therapeutic misconception".

Finally, while article 18 of the CRC provides that ‘States Parties shall use their best efforts to ensure recognition of the principle that both parents have common responsibilities for the upbringing and development of the child,’ the same article specifies that ‘the best interests of the child will be their basic concern’ [5]. There is thus potential for conflict between, on the one hand, the responsibility (of parents, the state, and researchers) to protect the best interests of the child; and on the other hand, the child’s right to be heard and to participate in the decision-making process.

In *Mabon v Mabon* [21], an English case on the right (of an adolescent) to be heard in custody proceedings, the court is instructive on this conflict and on article 12 of the CRC. Lord Justice Thorpe writes ‘we must, in the case of articulate teenagers, accept that the right to freedom of expression and participation outweighs the paternalistic judgment of welfare’ [21]. This passage suggests that a broader interpretation of the BIC is emerging in light of article 12. An interpretation of the BIC that is restricted to an objective standard is no longer sufficient. Instead, the BIC must include space for the maturing child to exercise his or her rights as enshrined in the CRC.

Under the CRC, the implication for paediatric genomic research is that a maturing child has a say in whether he or she participates in research, and whether his or her results are to be returned. In some cases the child’s best interests are paramount, while in others, these interests may be overridden. This duality imposes unique responsibilities on researchers to ascertain the child’s wishes, the weight of these wishes relative to the child’s age and maturity, and the lengths to which paediatric participant rights must be safeguarded. Likewise, States Parties must consider these rights when designing legislative regimes surrounding paediatric genomic research.

#### Right to the enjoyment of the highest attainable standard of health

The ‘right of the child to the enjoyment of the highest attainable standard of health’ [5] is another important principle for the issue of return of research results in the field of paediatrics. Indeed, article 24(2) of the CRC requires that States pursue full implementation of this right and carry out appropriate measures: ‘…b) to ensure the provision of necessary medical assistance and health care to all children with emphasis on the development of primary health care’ [5].

The majority of case law on the child’s right to health pertains to immigration litigation, and is therefore largely irrelevant to paediatric research. That being said, in a 2003 General Comment on HIV/AIDS and the rights of the child, the UN Committee associated the child’s right to health with States Parties ensuring that ‘HIV/AIDS research programmes include specific studies that contribute to effective prevention, care, treatment and impact reduction for children’ [16].

While the UN Committee is currently drafting a *General Comment* on the child’s right to health, the Human Rights Council recently adopted a resolution [22] that briefly discusses this right. In article 37 of the *Resolution*, the Council calls upon states ‘to take all necessary measures to ensure the right of the child to life, survival and the enjoyment of the highest attainable standard of physical and mental health is promoted and protected’. To the extent that the decision to return research results that are clinically significant, useful and actionable during childhood can result in preventative and/or therapeutic treatment, a connection may be drawn between the right to the highest attainable standard of health and the return of research results. Though the right to the highest attainable standard of health is predicated upon clinical action, the decision to return such research results is sufficiently tied to clinical care that it merits theoretical inclusion. Indeed, a number of international norms on research generally have associated the return of results with the health and quality of life of participants [4].

### Regional norms

In Europe, children’s rights are increasingly grounded in international principles (such as those discussed above), and as a result, they shed some light on the issues of paediatric research, return of results and the right not to know.

#### The best interests of the child

The *European Convention on Human Rights* [23], a treaty aimed at protecting human rights and fundamental freedoms in Europe, does not specifically address the rights of children. Consequently, the European Court of Human Rights (‘ECtHR’) must increasingly refer to the CRC and interpretations thereof in cases involving children [24].

In a landmark custody case on the interpretation of the BIC, the ECtHR described the BIC as a principle requiring primary consideration in decision-making concerning children.^c^ The Court added that the principle could, depending on the nature and seriousness of the situation, override parental interests. While the Court decided that parental interest remains a factor when balancing the various interests at stake, it insisted that this does not entitle parents to take measures that would harm the child’s health and development. The BIC ‘will depend on a variety of individual circumstances, in particular his age and level of maturity, the presence or absence of his parents and his environment and his experiences’ [25]. In addition, when adjudicating a case concerning children, the Court must ‘conduct an in-depth examination of the entire family situation and of a whole series of factors, in particular of a factual, emotional, psychological, material and medical nature, and make a balanced and reasonable assessment of the respective interests of each person, with a constant concern for determining what the best solution would be for the (…) child’ [25].

Although, *prima facie*, parental custody is a world apart from genetic research, the BIC principle is also applicable to the return of results in genomic research. In point of fact, according to Ross LF et al.:

In deciding whether a child should undergo [genetic testing], the focus must be on the child’s medical best interests; however, parents and guardians may also consider the potential psychosocial benefits and harms to the child and the extended family. Extending consideration beyond the child’s medical best interest not only acknowledges the traditional deference given to parents about how they raise their children, but also recognizes that the interest of a child is embedded in and dependent on the interests of a family unit [26].

Thus, the BIC framework sits in "[…] a web of moral, legal, medical or social policies about duties to people who cannot make decisions for themselves, including […] acceptable thresholds of care" [14]. Consequently, researchers must not only defer to the BIC principle in the context of return of results, but must also evaluate this principle in light of competing interests. In Europe, this deference entails an assessment of the multiple factors identified by the ECtHR, where the weight attached to each factor necessarily depends on the context. Nevertheless, in such analysis, considerations other than the BIC should be considered. We now turn to these.

#### The Child’s right to be heard: autonomy vs. Parental authority

The child’s right to autonomy is often juxtaposed with parental rights (*i.e*. ‘parental authority’). Interestingly, both of these rights are derived from the same source -- article 8 ECHR on the ‘[r]ight to respect for private and family life’ [23]. Other conventions have also addressed these rights. Chapter II (articles 5 to 9) of the *Convention on Human Rights and Biomedicine* (CHRB) [27] *,* also known as the *Oviedo Convention*, addresses the issue of informed consent. In the case of a minor who lacks capacity, article 6(2) states that consent can only be given by an authorised representative and that the opinion of the minor ‘shall be taken into consideration as an increasingly determining factor in proportion to his or her age and degree of maturity’.

Similar to the UN Committee’s *General Comment* on the right to be heard, the *Explanatory Report* to the CHRB mentions that ‘in certain situations which take account of the nature and seriousness of the intervention as well as the minor’s age and ability to understand, the minor’s opinion should increasingly carry more weight in the final decision’ [28]. This is all the more pertinent given article 27 of the Council of Europe’s *Additional Protocol on Biomedical Research,* which reads:

If research gives rise to information of relevance to the current or future health or quality of life of research participants, this information must be offered to them. That shall be done within a framework of health care or counselling. In communication of such information, due care must be taken in order to protect confidentiality and to respect any wish of a participant not to receive such information [29].

According to the *Explanatory Report*, the word ‘offered’ implies that participants must consent to or refuse any future communication of information prior to participation in research. This indicates that participants have a choice when the results become available.

Several cases on the interpretation of the European instruments described above have explored the interplay between the child’s right to autonomy and parental authority. Unfortunately, none of these cases specifically address the issue of research. However, given the fact that research is intended to produce generalizable results for society as a whole, the bar for return of results in this context is arguably higher than in the context of clinical care, where doctor-patient interests are aligned and treatment is intended to provide therapeutic benefit to individual patients.

The early 1988 ECtHR case of *Nielsen v Denmark* [30] involved a child’s detention in a psychiatric hospital at his mother’s request.^d^ The key issue was whether this was a lawful exercise of the mother’s parental authority under article 8. The Court held that the ‘exercise of parental rights constitutes a fundamental element of family life’ [30]. However, the Court went on to add, ‘the rights of the holder of parental authority cannot be unlimited and that it is incumbent on the State to provide safeguards against abuse’ [30].

The *Nielsen* case is important because it strongly reaffirms the principle that parents are authorized to make healthcare decisions on behalf of their children. Although the Court recognized the existence of limitations to parental authority, it did not adequately define these limitations. A discussion on this topic was held nearly twenty years later in *R (on the Application of Axon) v Secretary of State for Health* [31].

*Axon*, a UK court decision, discussed the limits on parental rights as articulated in article 8 of the ECHR in terms of the child’s right to autonomy. The case involved a mother who challenged a UK health policy allowing physicians to prescribe birth control to adolescent women without informing parents. Upholding the policy, Justice Silberman said that parental authority ‘dwindles as their child gets older and is able to understand the consequence of different choices and then to make decisions relating to them’ [31].

In other words, the child’s right to autonomy increases with maturity and eventually trumps parental authority. This is because parental rights are, in reality, duties, and extend only so far as needed for parents to discharge their responsibilities relating to the child’s upbringing and development.^e^ As the child becomes more and more capable of guiding their own development, parental responsibilities naturally diminish. Herein is the limiting principle as alluded to by the ECtHR in *Nielsen.*

The implications of *Nielsen* and *Axon* for genomic research are unclear, but some potential themes emerge. While parents have authority to make decisions concerning their child’s participation in research and as concerns return of results, that authority ‘dwindles’ as the child matures. Thus the researcher should be conscious of the child’s increasing autonomy in decision-making and take steps to safeguard it where appropriate.

That being said, it is important to note that *Nielsen* was a case of psychiatric confinement and *Axon* involved the parents’ right to be informed as to their child’s use of birth control.

#### The right not to know

Article 10 of the CHRB concerns the right not to know information about one’s health.^f^ Article 10 states:

Everyone is entitled to know any information collected about his or her health. However, the wishes of individuals not to be so informed shall be observed.

In exceptional cases, restrictions may be placed by law on the exercise of the rights contained in paragraph 2 in the interests of the patient [27].

The right not to know can be restricted in certain circumstances when it is in the best interests of the patient or on an exceptional basis [27], as provided in article 26(1). As the *Explanatory Report* points out, these exceptions would allow for informing patients of a predisposition to disease if this information could enable individuals to take ‘potentially effective (preventive) measures’ [28], even if the person has expressed the wish not to know this information. Similarly, ‘the possibility for prevention of the risk to [a] third party might […] warrant his or her right taking precedence over the patient’s right to privacy, […] and as a result the right not to know’ [28]. Again, the BIC is a primary consideration, but not the only consideration. In such cases, the Explanatory Report suggests that internal law balance the rights. As will be explored in Section 4, some European countries have chosen to enact legislation allowing an exception to privacy in the context of medical research whenever there is a serious risk to the health or life of a family member.

As mentioned earlier, article 27 of the *Additional Protocol* stipulates that information of relevance to current or future health or quality of life must be offered to the participants themselves, and that any wish not to receive such information must be respected as part of the duty of care [29]. This article, however, does not address the parent–child relationship, as parents can refuse to receive information pertaining to their children’s health.

Nevertheless, professional societies such as the European Society of Human Genetics (‘ESHG’) have adopted more specific guidance, providing that ‘parents are responsible to inform their children about their genetic risks’ [32]. Indeed, a 2012 ESHG *Principles for Good Practice in Paediatric Biobanks* goes further: ‘[t]he right of parents to receive or not receive genetic information about their children is limited’ [33]. The limited nature of parental authority mirrors the *Axon* case [31] where the child’s developing autonomy reached a tipping point at which it trumped the parental right to know. Interestingly, the ESHG adds that ‘[i]n the rare case that information about a preventable or treatable early-onset disease is found, [parents] *should* be notified regardless of their wishes providing the findings are subject to assessment of clinical validity and utility’ [33] (emphasis added). This more specific guidance on the return of results in the paediatric context protects the child’s best interests in the context of health care and research, and is echoed in the positions of several countries.

Interestingly, the *Whole Genome Sequencing in Health Care: Recommendations of the European Society of Human Genetics* [34] is not only concerned with the child’s right to autonomy, but also with the preservation of his or her future autonomy in the context of return of results. Specifically, the ESHG is concerned that the child’s right to be informed (or to refuse information) will be undermined as he or she matures. However, the *Recommendations* also stress that there must be ‘balance’ between the child’s interests and autonomy ‘and the parental rights and needs (not) to receive information that may be in the interest of their (future) family’ [34]. Finally, some authors have suggested that research results should be discussed during the informed consent process and that researchers have a duty to return results when the criteria of scientific validity, clinical utility, and medical actionability are met [35]. The parents’ right not to know cannot be used to override the duty owed to the child.

### National norms^g^

#### France

In France, parents are vested with decision-making authority on all matters relating to health care until such time as children reach the age of majority (i.e., eighteen years of age). Parents are responsible for the health and security of their children, and under French law, parental authority must always be exercised in the best interests of the child [36]. Moreover, since the ratification of the CRC by the French Parliament in 1990, parental decision-making must also respect the fundamental principles outlined therein.^h^

The *Code de la santé publique* [37] has detailed a number of provisions relating to the participation of children in biomedical research. While consent must be given by both parents [37],^i^ children must be consulted and their views taken into account, according to their level of maturity [37] and in conformity with the right to be heard, as found in the CRC. Accordingly, section L-1122-2 of the CSP requires researchers to obtain paediatric consent and to respect the child’s consent or refusal, as the case may be.

The new *Loi Jardé* [38] (adopted but not yet in force) specifically addresses the return of research results. These new provisions would mandate the provision of feedback during baseline assessment (*i.e.,* upon recruitment) (*Loi Jardé* L1121-1) as well as feedback of general research results (*Loi Jardé* L1121-1). Moreover, participants have a right to information, in the course of and at the conclusion of the research project, concerning their health, in the form of a written document given to individuals who have consented to receive said information (*e.g*. guardians/parents) [38]. Section L1131-1-1 also stipulates that donors who participate in secondary genetic research can consent (or refuse) to be informed of serious genetic anomalies.

Although parents are responsible for consenting to the return of individual research results concerning the health of their children, they can also refuse to be informed, as long as their refusal respects the requirement of section 371–1 of the *Civil Code* – *i.e*., that this decision be in the best interests of the child. In any case, clinician-researchers have a duty to protect the best interests of the child [37, 39], and as a consequence, may choose to implement policies that would see the return of paediatric results even where parental refusal has already been exercised [38].

#### United Kingdom

In the UK, the 1985 *Gillick* case [40] is a clinical and jurisprudential landmark for the principle that the capacity of a minor to consent to medical care should be determined on a case-by-case basis. According to the majority of the Court, it must be determined whether the child has sufficient understanding and intelligence to fully comprehend what is involved in a proposed intervention.

In the UK, the statutory age of presumed maturity (in the context of medical care) is age sixteen [41]; however, this age may be lower in particular treatment situations. It is important that the age of consent for medical care not be confused with the age of consent for research, including the choice to receive (or not) research results and incidental findings. Moreover, it cannot be presumed that ethics review committees and UK courts would judge that the threshold for capacity to consent to research is the same for treatment, especially considering the risks and benefits of the latter.

When children are deemed to be incapable of consent, parents (or legal guardians) must consent to health care or medical research on behalf of their children. In such cases, parents are presumed to have acted in the best interests of their children [42]. The BIC is determined according to children’s wishes, their capacity to understand their options and the consequences of these options, their emotional and physical needs, their parents’ wishes and the need to minimise restrictions on future autonomy, among other factors [42, 43]. Children should be involved as much as possible in the decision-making process and their views should be taken into account.^j^ Where parents and health professionals disagree on what is in the best interests of the child, the health professional may seek the opinion of the court. In such cases, the courts have traditionally followed the opinion of the physician [44]. In *Re MM (Medical Treatment)* [45], for example, the parents of a child suffering from primary immunodeficiency requested that their son continue with a programme of immunostimulant therapy and not receive replacement immunoglobin intravenously, as suggested by his physicians. Although the parties eventually came to an agreement during the course of the hearing, the Court nonetheless laid out the extent to which it would have been prepared to intervene in this case:

Although the parents’ objections to the particular treatment recommended by the doctors were rational objections, the court would have been prepared to override them had that been necessary, because the evidence that this treatment was in the child’s best interests was overwhelming [45].

In 1994, the Clinical Genetics Society issued a report on the genetic testing of children [46]. Though this report was not specifically aimed at genetic research, it recommended that genetic testing be limited to childhood onset disorders where useful medical intervention exists. In contrast, testing for adult-onset disorders and carrier status (having reproductive significance only) should generally be deferred until adulthood. The recommendations required that any decisions concerning immediate testing or deferral should be made on the basis of the best interests of the child, and should also involve a discussion between the parents and genetics health professionals.

Recent guidance from the British Society for Human Genetics on whole genome sequencing limits itself to questioning whether UK law would ‘restrict evidence of best interests to a narrow clinical context’ [44]. The PHG Foundation’s 2011 review of the implications of whole genome sequencing states that increased involvement of minors in decision-making through assent is standard practice, and identifies re-consent at age of majority as an emerging practice [47]. Finally, as in France, medical deontology in the United Kingdom dictates that parental refusal should be overridden in cases where the communication of health information would be in the best interests of the child [44].

#### Spain

Similarly to the *Loi Jardé* in France, the Spanish *Law 14/2007 of 3 July 2007 on Biomedical Research* contains provisions specific to the communication of research results. Section 4(5) states that everyone has a right to be informed of his or her genetic data or other data of a personal nature obtained during the course of research. This same right exists for retrospective (secondary) tissue research. The corollary of this right is an obligation for researchers to inform participants of relevant health information arising from research [48]. That being said, participants could refuse to receive such information in conformity with the 1997 *Oviedo Convention* by exercising their right not to know under article 4(5) of the 2007 law, which states that:

There shall be an observance of the right of the person not to know that data, which is referred to in the former paragraph, including unexpected findings that could arise. Nonetheless, when this information, according to the criteria of the doctor in charge, is necessary in order to avoid serious damage to his health or that of his biological family members, a close family member or a representative shall be informed, after consulting with the clinical ethics committee, if it exists [48].

In short, while no specific mention is made of minors, it would seem that parental refusal could also be overturned if certain legal conditions were met.

#### The Netherlands

The *Medical Research Involving Human Subjects Act* governs research in the Netherlands [49]. According to section 4, the legal age of consent is 18. The participation of a minor (under the age of 18) in research would require the written consent of parents or legal representatives, if applicable [49].

As for the issue of return of research results, there are no legal provisions on the communication of these results in Dutch law. That said, the 2011 *Code Goed Gebruik* of the Federation of Dutch Medical Scientific Societies recommends that results be returned where: 1) there is a real risk of a serious condition; 2) a professionally-recognised treatment can be offered to the donor; 3) the donor is known to carry the risk factor; 4) disclosure would allow treatment of his or her condition; and, 5) the donor has consented [50]. Similar to the Spanish *2007 Law on Biomedical Research,* the Dutch *Code of Conduct* also recommends the return of results if doing so might benefit a family member.

#### Canada

The second edition of the *Tri-Council Policy Statement* (TCPS 2) – a pan-Canadian normative document governing research – requires that research ethics committees ensure, among other things, that researchers seek and maintain ‘consent from authorized third parties in accordance with the best interests of the persons concerned’ [51], when individuals who lack the capacity to consent are participating in research. In Canada, the definition of the BIC principle is statutorily enshrined in provincial legislation. In the province of Quebec [52], for example, Article 33 of the *Civil Code of Quebec* states: "Every decision concerning a child shall be taken in light of the child's interests and the respect of his rights. Consideration is given, in addition to the moral, intellectual, emotional and physical needs of the child, to the child's age, health, personality and family environment, and to the other aspects of his situation" [53]. In British Columbia, Article 37(2) of the *Family Law Act* stipulates that, concerning guardianship:

To determine what is in the best interests of a child, all of the child's needs and circumstances must be considered, including the following: (a) the child's health and emotional well-being; (b) the child's views, unless it would be inappropriate to consider them; (c) the nature and strength of the relationships between the child and significant persons in the child's life; (d) the history of the child's care; (e) the child's need for stability, given the child's age and stage of development; (f) the ability of each person who is a guardian or seeks guardianship of the child, or who has or seeks parental responsibilities, parenting time or contact with the child, to exercise his or her responsibilities; (g) the impact of any family violence on the child's safety, security or well-being, whether the family violence is directed toward the child or another family member; (h) whether the actions of a person responsible for family violence indicate that the person may be impaired in his or her ability to care for the child and meet the child's needs; (i) the appropriateness of an arrangement that would require the child's guardians to cooperate on issues affecting the child, including whether requiring cooperation would increase any risks to the safety, security or well-being of the child or other family members; (j) any civil or criminal proceeding relevant to the child's safety, security or well-being [54].

Last, in Manitoba, Article 2(1) of *The Child and Family Services Act* holds that:

The best interests of the child shall be the paramount consideration of the director, an authority, the children's advocate, an agency and a court in all proceedings under this Act affecting a child, other than proceedings to determine whether a child is in need of protection, and in determining best interests the child's safety and security shall be the primary considerations. After that, all other relevant matters shall be considered, including (a) the child's opportunity to have a parent–child relationship as a wanted and needed member within a family structure; (b) the mental, emotional, physical and educational needs of the child and the appropriate care or treatment, or both, to meet such needs; (c) the child's mental, emotional and physical stage of development; (d) the child's sense of continuity and need for permanency with the least possible disruption; (e) the merits and the risks of any plan proposed by the agency that would be caring for the child compared with the merits and the risks of the child returning to or remaining within the family; (f) the views and preferences of the child where they can reasonably be ascertained; (g) the effect upon the child of any delay in the final disposition of the proceedings; and (h) the child's cultural, linguistic, racial and religious heritage [55].

In *AC v Manitoba (Director of Child and Family Services),* the Supreme Court of Canada further described the BIC as operating according to a:… sliding scale of scrutiny, with the child’s views becoming increasingly determinative depending on his or her maturity. The more serious the nature of the decision and the more severe its potential impact on life or health, the greater the degree of scrutiny required […] The more a court is satisfied that a child is capable of making a truly mature and independent decision on his or her own behalf, the greater the weight that must be given to his or her views when a court is exercising its discretion […] If, after a careful analysis of the young person’s ability to exercise mature and independent judgment, the court is persuaded that the necessary level of maturity exists, the young person’s views ought to be respected [56].

As for the return of individual findings to participants, the TCPS 2 created an ethical obligation for researchers to return what it describes as ‘material’ incidental findings – or ‘findings interpreted as having significant welfare implications for the participant, whether health-related, psychological or social’ [51]. According to the TCPS 2, researchers are also required to develop a plan for managing information that may be revealed through their genetic research, submit such a plan to their research ethics committees, and advise prospective participants of the plan to manage their information [51]. Participants should be provided with an opportunity to make informed choices on whether they wish to receive information about themselves, and be given the opportunity to express their preference on whether information emanating from the research project can be shared with biological relatives [51]. This preference, however, may be subject to ‘overriding considerations’ such as information on life-threatening conditions that ‘can be prevented or treated’.

The Centre of Genomics and Policy and the Mother Infant Child and Youth Network, in collaboration with the Ethics Office of the Canadian Institutes of Health Research, have adopted more specific guidance in their 2012 *Best Practices for Health Research involving Children and Adolescents* [4] *.* According to this document:

Generally, researchers should respect the wishes of the competent adolescent, or those of the parents, if the minor is not competent to consent, regarding the return of research results. However, individual results and incidental findings should be communicated 1) if they are scientifically valid, 2) if they have significant implications for the health of the child or adolescent, and 3) if there is a means of prevention or treatment available during childhood or adolescence [4].

The *Best Practices* later specify that ‘when the research involves school-age minors capable of assent, the information should also be delivered to them with the agreement of their parents, and in a manner appropriate to their development, level of understanding and degree of maturity’ [4].

### Rights, responsibilities and the best interests of the child

The foregoing discussion has raised several questions on the meaning of the BIC and its relation to other laws and rights, both under the CRC and elsewhere. Is it in the best interests of the child to allow him or her to ‘subjectively’ choose a course of action that may result in adverse or suboptimal consequences? Or is it in the child’s best interests to impose an ‘objectively’ favourable course of action that would override his or her wishes? In such cases, how should the right to be heard factor into the decision-making process?

Some have accused the BIC principle of being overly paternalistic and have questioned whether it is, in fact, at odds with children’s rights and respect for their autonomy [8]. Consider the comments of Diana Bryant, Chief Justice of Australia’s Family Court:

If children’s immutable rights are in issue we should say so, rather than attempting, as I think we have done, to quietly subsume human rights considerations under the rubric of ‘best interests’ [8].

There is thus a growing appreciation of the fact that a careful balance must be struck between adequate welfare protection of a still-developing child and the child’s other rights – in particular, his or her right to autonomy and self-determination with due regard for age and maturity. That being said, at what point does the opinion of the minor become the only opinion that should be taken into account [57]? The answer to this question can only be determined on a case-by-case basis.

In contrast, other commentators have called the BIC overly individualistic, arguing that ‘a child’s interests cannot be completely distinguished from those of his or her parents, but are always intertwined with those of the parents and siblings’ [58]. Interests other than those solely of the child can, and should, come into play where decisions affecting the child may potentially impact the family unit. The child’s interests are intimately connected with those of his or her family – a fact that should not be ignored.

Others have argued that the child’s right to be heard in article 12 of the CRC should be understood as a ‘threshold’ requirement [59]. Once the child is mature enough to make a decision on any matter affecting his or her interests, he or she has the right to do so. Parental authority cannot be used to outweigh the child’s autonomy once that threshold of maturity has been reached (*i.e*., a *Gillick*-like test on a case-by-case basis).

This issue raises a further question: whose responsibility is it to ensure the protection of paediatric rights? Whereas in the past, parents were viewed as ‘custodians’ [10] of paediatric rights, with the advent of the CRC, the scope of actors sharing in that responsibility and their *marge de manoeuvre* was substantially widened. Commenting on the advent of the CRC, Reading et al. write:

One of the far-reaching consequences of the [CRC] is that it makes the child an individual with rights and not just a passive recipient, and hence the child has the right to actively participate at all levels of decision-making. The traditional association between the state, the family, and the child could be conceptualised as a series of concentric circles with the child at the centre. The [CRC] implies that this association should now be understood to be triangular in which the state has a direct responsibility to the child to promote her or his rights [60].

To this triangular structure, we suggest adding a fourth actor – the researcher – in the context of paediatric research. Indeed, the researcher’s responsibilities to the child participant could be said to exist independently of those of the parents or State.^k^ This new structure raises questions as to the nature of State and researcher responsibilities towards the child and how far they must go in discharging those responsibilities. As demonstrated, different regional and national norms have approached these issues in different ways.

Genomic research and the question of return of results in the paediatric context give rise to new challenges for the BIC principle and the rights of the child. The arrival of next-generation sequencing has challenged whether the BIC principle and the child’s ‘anticipatory’ autonomy rights or ‘right to an open future’ (whereby decision-making concerning genetic screening of adult-onset disorders must be deferred until adulthood) continue to offer a suitable (or even workable) framework [47, 61]. Likewise, by examining the entire human genome (as opposed to discrete sections), whole genome sequencing has changed the nature of genetic testing and increased the possibility of incidental findings, thereby potentially pre-empting the child’s right to defer test-related decisions until adulthood [47, 61, 62].

With this in mind, it is interesting to note the controversy surrounding the more radical position of the American College of Medical Genetics and Genomics (ACMG) in its *Recommendations for Reporting of Incidental Findings in Clinical Exome and Genome Sequencing*, published on 19 March 2013 [63]. While an in-depth discussion is beyond the scope of this paper, the *Recommendations* mandated that laboratories test for 57 reportable genetic conditions during whole genome sequencing, irrespective of the ages of participants or their wishes. Shortly thereafter, the ACMG published a clarification re-affirming this position and imposing an overall obligation to report pathogenic variations for these 57 genes irrespective of choice [64]. In justifying this, the ACMG pointed to the potential implications for parents in cases where actionable genetic predispositions are discovered through genomic sequencing of related children (*e.g*. in cases concerning the BRCA1 gene). The ACMG’s perspective challenges the BIC in favour of a broader, more family-centric view, and seemingly brings the parents’ interests in direct conflict with those of the child.

## Summary

Our comparative analysis of international, regional and national norms pertaining to the return of research results has revealed a dearth of normative documents specifically addressing the issue of return of results in paediatric research. Though the best interests of the child principle guides both international and regional normative paradigms, other principles, such as the right to autonomy, the right to be heard, the right to enjoy the highest attainable standard of health and the right not to know, further delimit the parameters of paediatric-specific approaches to the question of return of research results in paediatric genomic research. Today, the challenge is to determine the content and scope of the BIC framework in the context of the increasingly recognised right to autonomy. The child’s future autonomy (upon reaching the age of maturity) is also an important consideration. Nevertheless, genomic research raises the possibility that the child’s parents may also have an interest in the research results, an issue that must likewise be considered.

National guidelines and laws differ in their approach to the return of results as well as on the role and obligations of the various stakeholders involved in the decision-making process. However, a trend towards making the return of results obligatory if this return is in the best interests of the child (during childhood) is clearly tangible. Moreover, based on laws and jurisprudential interpretations, parental refusal of results may be overridden if doing so would be in the child’s best interests. Given that next-generation sequencing technologies may reveal clinically significant health information of importance to the child prior to maturity, the duty to protect children from harm and promote their health interests lends to the application of a more proactive approach, one that would mandate return irrespective of parental preferences under certain conditions. Return of paediatric results, however, is not akin to the duty to actively seek out or hunt for findings.

Finally, given that research is increasingly international and collaborative, initiatives fostering discussion between members of the paediatric research community should be encouraged (see for example the position on the return of results of the International Paediatric Research Platform of the Public Population Project in Genomics and Society [65]). Indeed, the sharing of policies, tools and ideas at the international level will not only ensure the efficient mobilisation of knowledge, but will also set the stage for uptake by the paediatric community to benefit those who need it most – the children.

## Endnotes

^a^For a review of US policies, see ‘Predictive Genetic Testing of Children and the Role of the Best Interest Standard’ [66]. For a review of US law, see ‘The Legal Risks of Returning Results of Genomics Research’ [67].

^b^The United Nations Committee on the Rights of the Child defines maturity as ‘the capacity of the child to express his or her views on issues in a reasonable and independent manner’ [11].

^c^The case of *Neulinger and Shuruk v Switzerland* [25] was brought by a mother who had unlawfully removed her son from Israel in 2005 and returned to her native Switzerland. The father sought the return of his son despite having shown a lack of interest in the boy. The mother argued that she could not return her son to Israel because she feared the risk of prosecution and conviction for the abduction. Her son would therefore have to go back alone. The mother argued that the father had not seen his son in the last two years and that returning the son would not be in the best interests of the child. The Grand Chamber of the Court considered that since the boy had been in Switzerland during the proceedings (7 years) and was well integrated, it would not be in his best interests to be returned to Israel without his mother [68].

^d^A mother sought confinement of her 12 year old son in a psychiatric hospital because ‘it was clear he did not want to live with her’ and the father did not have custody rights. The hospital accepted the mother’s wishes as the holder of parental authority despite the lack of an adequate diagnosis. The child challenged the lawfulness of the confinement through his father.

^e^*Axon* confirmed this principle from the earlier English case *Gillick v West Norfolk and Wisbech Area Health Authority* [40] which, although similar to *Axon*, did not refer to the ECHR. See discussion of the United Kingdom for an examination of the *Gillick* case.

^f^See also Chapter VII of the *Additional Protocol* [29]. The *Explanatory Report on the Convention* mentions that ‘a person’s "right to know" encompasses all information collected about his or her health, whether it be a diagnosis, prognosis or any other relevant fact’ [28].

^g^This section is based on BM Knoppers, A Rioux and MH Zawati [3].

^h^Treaties that have been ratified by the French Parliament are automatically integrated in domestic law. These treaties are in theory considered to be hierarchically superior and to prevail over domestic legislation in accordance with section 55 of France’s Constitution of 4 October 1958.

^i^However, if the research project meets three conditions: 1) negligible risks, 2) the minor is not participating to research as a healthy volunteer and 3) it is impracticable to obtain the other parent’s consent in time, the consent of one of the parents is sufficient

^j^The involvement of the child appears to be an important principle as it is mentioned in most laws and guidelines [69].

^k^While on paediatric oncology, on this point see Hens [33, 57].
